# Supplementary material for: The Ligand Binding Domain of the Cell Wall Protein SraP Modulates Macrophage Apoptosis and Inflammatory Responses in Staphylococcus aureus Infections
Source: Molecules. 2025 Mar 5;30(5):1168. doi: 10.3390/molecules30051168 (PMC11901804; doi:10.3390/molecules30051168)
Supplement: Supplementary file 1 [file molecules-30-01168-s001.zip › molecules-3483749-supplementary.pdf]

Supplementary materials

The Ligand Binding Domain of the Cell Wall Protein SraP Modulates Macrophage Apoptosis and Inflammatory Responses in *Staphylococcus aureus* Infections

He Sun, Robert W. Li, Thomas T. Y. Wang and Lin Ding

Figure S1. The cell wall protein serine rich adhesin for platelets (SraP) L-lectin module (LLM) mRNA expression in the wild-type strain harboring an empty expression vector (pRMC2) and LLM overexpression (pRMCL-lectin) detected using quantitative RT PCR. **** $p < 0.0001$.

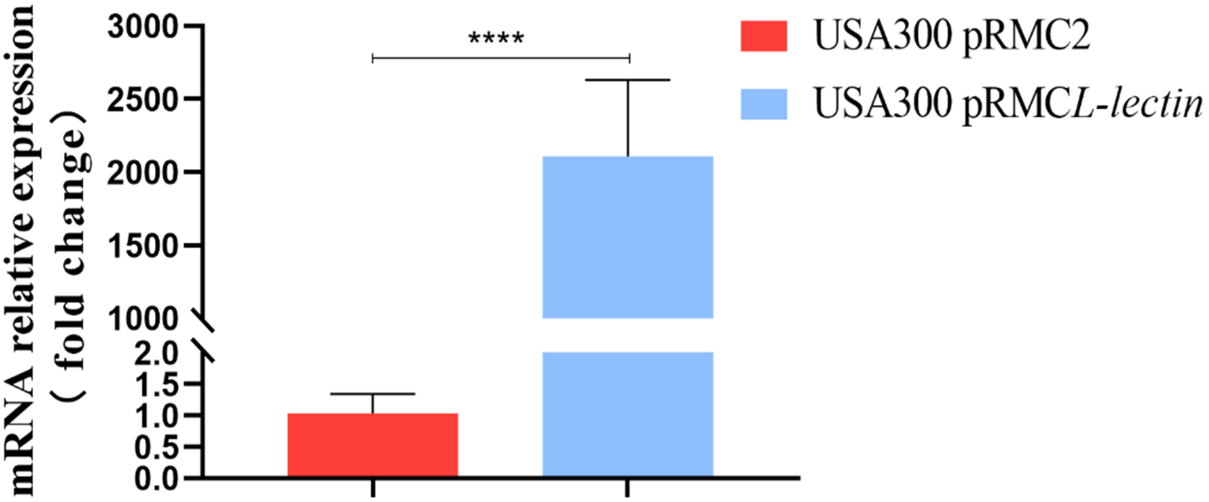

Table S1. Primers used in the study.

Primer ID	Primer sequence (5'-3')
<u>Primers used for the construction of LLM deletion and overexpression mutants</u>	
L-lectin-up-F	AGGTGGATGGTATAGATATATTA
L-lectin-up-R	TTGTGTAACAGCAGACTCTGTTGACATAGCTAAGCGACTG
L-lectin-down-F	CGCTTAGCTATGTCAACAGAGTCTGCTGTTACACAAGTGA
L-lectin-down-R	TTGATACACTATTACTTTCAGAT
L-lectin-pKOR1-F	TGAGCCTCGGAACCGGTACCATTCACGGTAAATATGTTGCA
L-lectin-pKOR1-R	GGCAGTGAGCGCAACGCAATTTGATACACTATTACTTTCAGA
pKOR1-L-lectin-F	GTAATAGTGTATCAAATTGCGTTGCGCTCACTGCCCGCTT
pKOR1-L-lectin-R	ATATTTACCGTGAATGGTACCGGTTCCGAGGCTCAACGTC
L-lectin-JD-F	AACAGCCGATTCCACAAGTGTAA
L-lectin-JD-R	TGAATTCGTTGCACTATCGTAAC
L-lectin-ter-F	TTTGCGTCAGCAGCGACGACA
L-lectin-ter-R	GTAAATTTGTCGCGCCACCTG
<u>Primers used for quantitative RT PCR of human genes</u>	
IL1B F	CCTGTCCTGCGTGTTGAAAGA
IL1B R	GGGAACTGGGCAGACTCAAA
IL6 F	CCTTCGGTCCAGTTGCCTTCT
IL6 R	GAGGTGAGTGGCTGTCTGTGT
IL10 F	TCTCCGAGATGCCTTCAGCAGA
IL10 F	TCAGACAAGGCTTGGCAACCCA
BAX F	CCCGAGAGGTCTTTTTCCGAG
BAX R	CCAGCCCATGATGGTTCTGAT
BCL2 F	GGTGGGGTCATGTGTGTGG
BCL2 R	CGGTTCAGGTACTCAGTCATCC
NLRP3 F	AACAGCCACCTCACTTCCAG
NLRP3 R	CCAACCACAATCTCCGAATG
IL8 F	ACATACTCCAAACCTTTCCACCC
IL8 R	CAGCCCTCTTCAAAAACTTCTCC
TGFB F	TACCTGAACCCGTGTTGCTCTC
TGFB R	GTTGCTGAGGTATCGCCAGGAA
NFκB F	GCAATCATCCACCTTCATTCTCAAC
NFκB R	CTCAGCAAATCCTCCACCACATC
AP1 F	TTCCTGCGTCTTAGGCTTCTCC
AP1 R	CTTCTTCTCTTGCGTGGCTCTC
CD64 (FcγRI) F	AGTTGATGGGCAAGTGGACACC
CD64 (FcγRI) R	GGCAGATGGAGCACCTCACAG
CD16 (FcγRIII) F	AAACCCTGTGTCACTGTCCCAAG
CD16 (FcγRIII) R	GCTTTCCTTCCTGTGTGCTTGTG
FAS F	ACAGCCACATTCCCGAGTTGAG

FAS R	ACCCAGTATGCCCACCACAAAG
GAPDH F	ACAACTTTGGTATCGTGGAAGG
GAPDH R	GCCATCACGCCACAGTTTC

Table S2. Relative mRNA expression of select transcription factors in macrophages infected with WT and LLM deletion strains, respectively.

Gene	ΔL-Lectin/WT Fold change	*p* value
STAT1	1.06±0.16	0.3859
STAT2	0.82±0.13	0.2148
STAT6	1.19±0.83	0.3485
IRF3	1.03±0.28	0.4552
IRF5	1.04±0.34	0.3245
JAK1	1.02±0.21	0.1157
PI3K	1.02±0.26	0.2968
